# D609 Suppresses Antituberculosis Response by Regulating Dendritic Cells Antigen Presentation

**DOI:** 10.1002/iid3.70103

**Published:** 2024-12-18

**Authors:** Honglin Liu, Huimin Huang, Zhen Huang, Yingxuan Chen, Deyou Tan, Xiaoni Wang, Xiaoni Pang, Shuwen Chen, Lianhui Liang, Haihui Yang

**Affiliations:** ^1^ Department of Clinical Laboratory Zhongshan Second People's Hospital Zhongshan Guangdong China; ^2^ Institute of Molecular Immunology, School of Laboratory Medicine and Biotechnology, Southern Medical University Guangzhou Guangdong China

**Keywords:** antigen presentation, D609, dendritic cells, *Mycobacterium tuberculosis*

## Abstract

**Objective:**

To elucidate the role of phosphatidylcholine‐specific phospholipase C (PC‐PLC) in the antituberculosis (anti‐TB) immune response mediated by dendritic cells (DCs).

**Methods:**

In vivo, C57BL/6J mice infected with the *Mycobacterium tuberculosis* strain H37Rv. Before infection, the mice were pretreated with the PC‐PLC inhibitor D609. Bacillary loads in lung and spleen tissues were quantified through colony‐forming unit (CFU) assays. Hematoxylin and eosin (H&E) staining was performed to assess inflammatory infiltration and tissue damage. Levels of inflammatory mediators in peripheral venous blood were quantified using enzyme‐linked immunosorbent assays (ELISAs). Flow cytometry was employed to determine the proportions of conventional DCs (cDCs) and their subsets, cDC1 and cDC2, within lung, spleen, and lymph node tissues. In vitro, mouse bone marrow‐derived dendritic cells (BMDCs) pretreated with D609. The expression levels of chemokines and pro‐inflammatory cytokines were assessed via quantitative polymerase chain reaction (qPCR) and ELISA. BMDCs were loaded with H37Rv expressing red fluorescent protein (RFP‐H37Rv) or DQ‐OVA, and flow cytometry was utilized to analyze the impact of D609 on antigen phagocytosis and processing. Furthermore, flow cytometry was employed to evaluate the effect of D609 pretreatment on the expression levels of costimulatory molecules on BMDCs. The capacity of D609‐treated BMDCs to activate and proliferate T cells, as well as to induce interferon‐gamma (IFN‐γ) secretion, was assessed through a DC‐T cell coculture system.

**Results:**

In vivo analysis revealed that mice pretreated with D609 exhibited a marked increase in tissue bacterial load, enhanced inflammatory infiltration, and a reduction in pro‐inflammatory mediator expression in peripheral venous blood. There was a notable decrease in the number of cDCs in lung and lymph node tissues, with a pronounced reduction in cDC1 in the lungs and cDC2 in the lymph nodes. In vitro studies demonstrated that D609 pretreated BMDCs displayed a significant decline in inflammatory mediator production, antigen phagocytosis, and antigen processing capabilities, potentially due to altered expression of costimulatory molecules. Coculture experiments indicated that D609 pretreated BMDCs showed a substantial reduction in their ability to stimulate T cell activation, proliferation, and IFN‐γ secretion.

**Conclusion:**

Our findings suggest that PC‐PLC plays a critical role in the functionality of DCs, including the production of chemokines and pro‐inflammatory cytokines, migration to lymph nodes, and antigen presentation to T cells, which collectively contribute to T cell activation and effective clearance of Mycobacterium tuberculosis. Further investigation into the regulatory mechanisms of PC‐PLC in DCs may uncover novel therapeutic targets for the development of advanced anti‐TB treatments.

## Graphical abstract

Our results demonstrated that D609 has a direct influence on the intrinsic immune process and a regulatory effect on adaptive immunity, thereby contributing to TB immunity. Its inhibitory role in association with PC‐PLC represents immune targets for the development of new antituberculosis therapeutic regimens.

## 1 Introduction

Tuberculosis (TB) is a fatal disease caused by infection with the bacterium *Mycobacterium tuberculosis* (*M. tuberculosis*). It is one of the most perilous communicable diseases worldwide, causing 1.6 million deaths and afflicting around 2 billion individuals with latent TB infection, as reported by the World Health Organization (WHO) in 2022 [1]. *M. tuberculosis* penetrates the respiratory tract and primarily localizes in lung phagocytes, such as macrophages, dendritic cells (DCs), and neutrophils [2]. DCs, identified by their distinctive morphology, have been implicated in the uptake, processing, and presentation of antigens [3]. They are the exclusive antigen‐presenting cells (APCs) that activate the naïve T cells that function as a bridge linking innate and adaptive immunity. Tricyclodecan‐9‐yl‐xanthogenate (D609) exhibits antiviral and antitumor properties. The primary function of D609 is to inhibit phosphatidylcholine (PC)‐specific phospholipase C (PC‐PLC). Moreover, D609 is an inhibitor of sphingomyelin synthase (SMS). The inhibition of PC‐PLC and/or SMS greatly affects the lipid signaling molecules 1,2‐diacylglycerol (DAG) and/or ceramide. The inhibition of PC‐PLC and/or SMS affects the cell cycle, causing arrested proliferation and stimulated differentiation in numerous in vitro and in vivo studies [4].

D609 plays a crucial regulatory function in multiple pathologies and physiological processes. Treatment with D609, RAW264.7, microglia, and astrocytes increased G1‐phase cell accumulation, subsequently reducing the quantity of S‐phase cells. Research into immunotherapy for age‐related macular degeneration has determined that D609 is a powerful inhibitor of excessive reactive oxygen species (ROS), preventing severe oxidative stress‐induced mitochondrial damage in retinal pigment epithelial (RPE) cells, causing RPE cell protection [5]. In tumor therapy, D609 inhibits the growth of neural progenitor cells by reducing ERK‐mediated cyclin D1 expression with potential therapeutic effects in preventing tumor stem cell growth [6]. In addition, D609 could function as a target for tumor cell apoptosis by modulating the cytotoxicity caused by U937 cells when exposed to tumor necrosis factor (TNF) and anti‐Fas antibodies [7]. D609 increased FasL‐induced apoptosis in T lymphocytes via caspase‐dependent and caspase‐independent mechanisms by inhibiting the conversion of ceramide to complex sphingomyelin [8]. Furthermore, D609 treatment reduced the generation of pro‐inflammatory cytokines triggered by lipopolysaccharide‐mediated cellular TLR4 activation, decreasing CD14 protein levels, and ultimately alleviating the LPS‐induced pro‐inflammatory response [9]. D609 has been reported to inhibit the activity of both herpes simplex virus (HSV‐1) encoded protein kinase (US3 PK) and cytosolic protein kinase type C1, thereby restricting the replication of HSV‐1 by attenuating the phosphorylation of polypeptides in infected cells [7]. Treatment of RAW264.7 murine macrophage‐like cells with LPS‐activated induced nitric oxide synthase (iNOS) and elevated levels of TNF‐α. The complete inhibitory effect was noted after combining D609 with butanol. This finding suggests that PC‐PLC activity could partially mediate the activation of LPS on RAW264.7 cells [10].

PC‐PLC, a vital enzyme present in the plasma membrane, can convert phosphatidylinositol 4,5‐bisphosphate (PIP2) into two key signaling molecules, namely, inositol 1,4,5‐trisphosphate (IP3) and DAG. It has been linked to the ability of D609 to function as an antagonist against pro‐inflammatory cytokines. Both IP3 and DAG are involved in significant functions in different cellular processes and serve as precursors for the production of other vital signaling molecules [11]. These molecules are crucial bases for the several cellular pathways related to cellular communication. While PLC has been previously reported to participate in the replication of *M. tuberculosis*, as well as TB immunity in macrophages and neutrophils [12, 13, 14, 15], and can induce cytoskeletal rearrangements of DC2.4 cells during TB infection, it remains unclear whether it impacts the immune functions of DCs. We investigated the impact of the PLC inhibitor D609 on the antituberculosis immune response, our findings indicate that D609 reduces the number of DCs and their immune function, which in turn affects T‐cell activity.

## 2 Results

### 2.1 D609 Compromised the Immune Response Against *M. tuberculosis* in Mice

**Figure 1:**
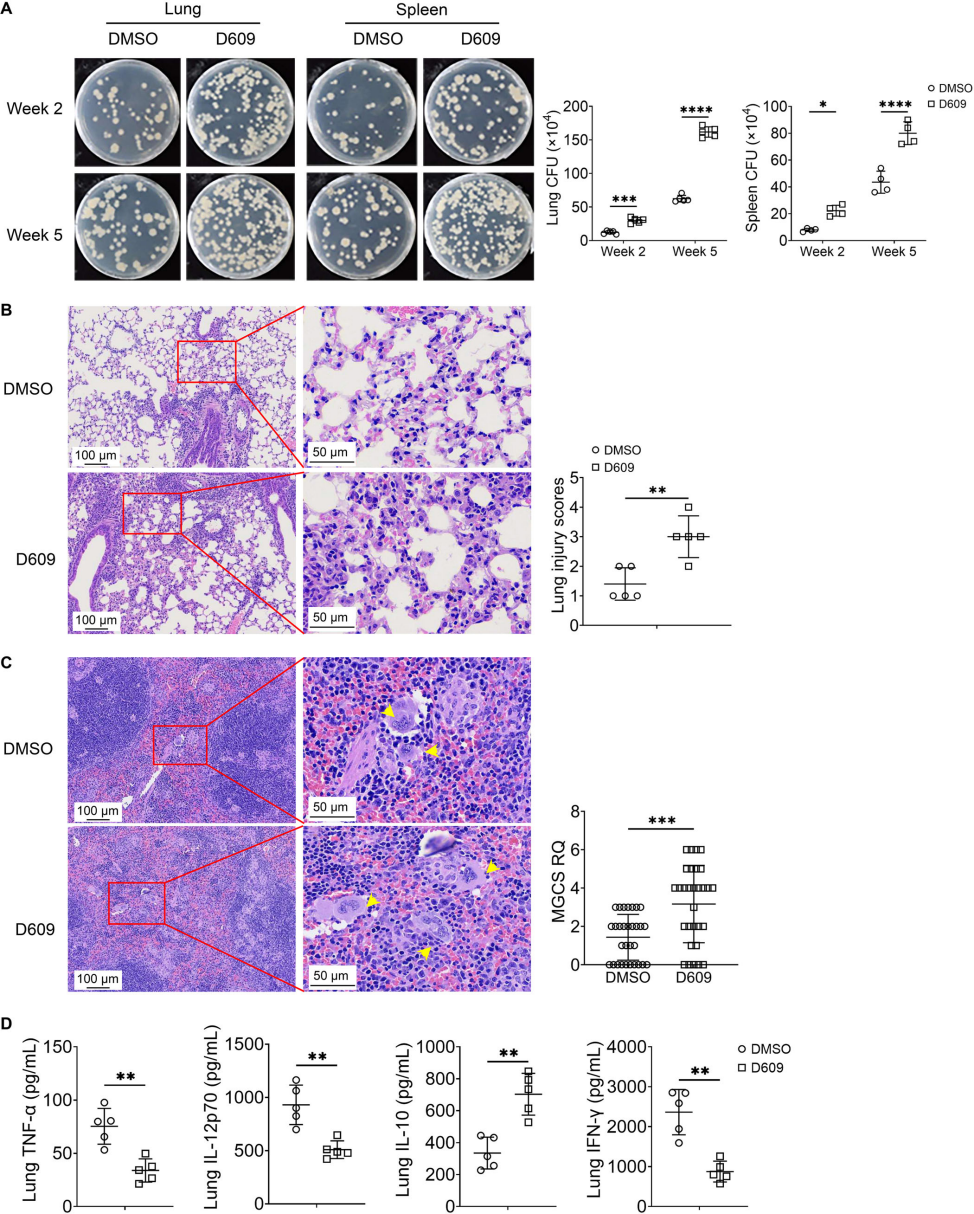
In vivo immune response of D609 treated mice infected with H37Rv. (A) In vivo H37Rv load in the lung and spleen of D609 (200 ng) treated mice at 2 and 5 weeks post H37Rv infection (*n* = 5). (B) The results of H&E staining and tissue score of the lung of D609 treated mice (*n* = 5，five randomly selected fields of view for tissue score statistics). (C) H&E staining of the spleen tissues of D609 treated mice. The amount of splenic MGCs was quantified (*n* = 5, 30 randomly selected fields of view for statistics). Yellow arrows indicate MGCs. (D) ELISA assays of cytokine expression in the lung of mice 2 weeks post H37Rv infection (*n* = 5). A two‐way ANOVA with Šidák's post hoc test (A) and an unpaired *t*‐test (B–D) were used for statistical analysis. Data are presented as mean ± SD and are representative of at least three experiments with similar observations. *p < 0.05, **p < 0.01, ***p < 0.001, ****p < 0.0001. ANOVA, analysis of variance; DMSO, dimethyl sulfoxide; ELISA, enzyme‐linked immunosorbent assay; H&E, hematoxylin and eosin; MGCs, multinucleated giant cells; SD, standard deviation.

To investigate the roles of D609 during *M. tuberculosis* infection, a model of *M. tuberculosis* infection was established by infecting mice with H37Rv, following a 1‐week period of infection with H37Rv, mice were injected intraperitoneally with D609 every 3 days. Bacterial load assessments in vivo revealed significantly higher levels of H37Rv in D609 mice than in DMSO mice (Figure 1A). Histological examination revealed that D609 treated resulted in marked tissue damage in the lungs, accompanied by inflammatory infiltration (Figure 1B). Moreover, we observed a significant increase in splenic multinucleated giant cells (MGCs), which is indicative of chronic inflammation (Figure 1C) [16]. The expression levels of cytokines TNF‐α, IL‐12p70, and IFN‐γ, which are associated with dendritic cell activation and T‐cell function, were reduced in the lung tissues of D609‐treated mice, while the expression of the inhibitory cytokine IL‐10 was increased (Figure 1D). In sum, these findings suggest that D609 plays a role in limiting the immune response against *M. tuberculosis*.

### 2.2 D609 Regulation of DC Cell Subtype Numbers and Immune Function

**Figure 2:**
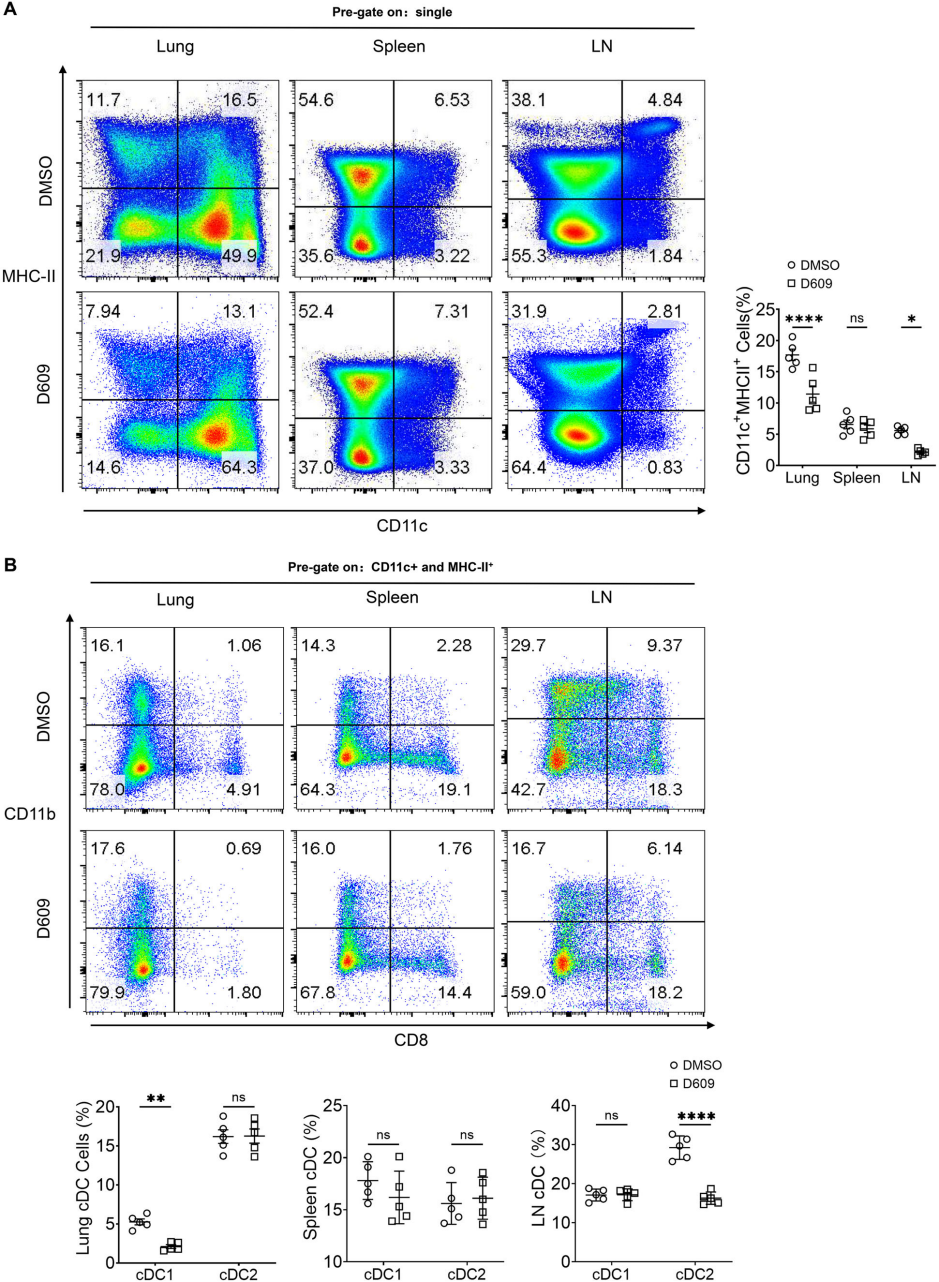
D609 modulation of dendritic cell subtype abundance in vivo. (A) Impact of D609 (200 ng) on dendritic cell abundance in the pulmonary, splenic, and inguinal lymph nodes (LN) microenvironment of mice 2 weeks post H37Rv infection. Quantification of conventional dendritic cells (cDCs) using flow cytometry analysis with CD11c and MHC‐II markers (*n* = 5). (B) Flow cytometry analysis of cDC1, cDC2 ratios using CD8 and CD11b markers within the cDC population (*n* = 5). A two‐way ANOVA with Šidák's post hoc test (A, B) was used for statistical analysis. Data are presented as mean ± SD and are representative of at least three experiments with similar observations. *p < 0.05, **p < 0.01, ***p < 0.001, ****p < 0.0001. ANOVA, analysis of variance; DMSO, dimethyl sulfoxide; SD, standard deviation.

It has been shown that D609 plays a key role in the anti‐*M. tuberculosis* process by inhibiting the function of PC‐PLC [14, 17]. PC‐PLC has been documented in macrophages, neutrophils, and even *M. tuberculosis* itself in the context of *M. tuberculosis*‐host immune interactions [12, 13, 14, 15]. However, the relationship between the PC‐PLC function of dendritic cells (DCs), another antigen‐presenting cell, and TB immunity has not yet been reported. Henceforth, we embarked upon a preliminary examination of the abundance of DCs in the pulmonary, splenic, and inguinal lymph nodes (LNs) regions of mice afflicted with H37Rv infection, and subsequently treated with D609. The findings derived from flow cytometry analyses exhibited a notable decline in the population of conventional DCs (cDCs) within the lung and LNs compartments of D609‐treated mice (Figure 2A). Notably, the pulmonary landscape was predominantly marked by a reduction in the cDC1 subset, whereas the LNs milieu was predominantly marked by a reduction in the cDC2 subset (Figure 2B).

**Figure 3:**
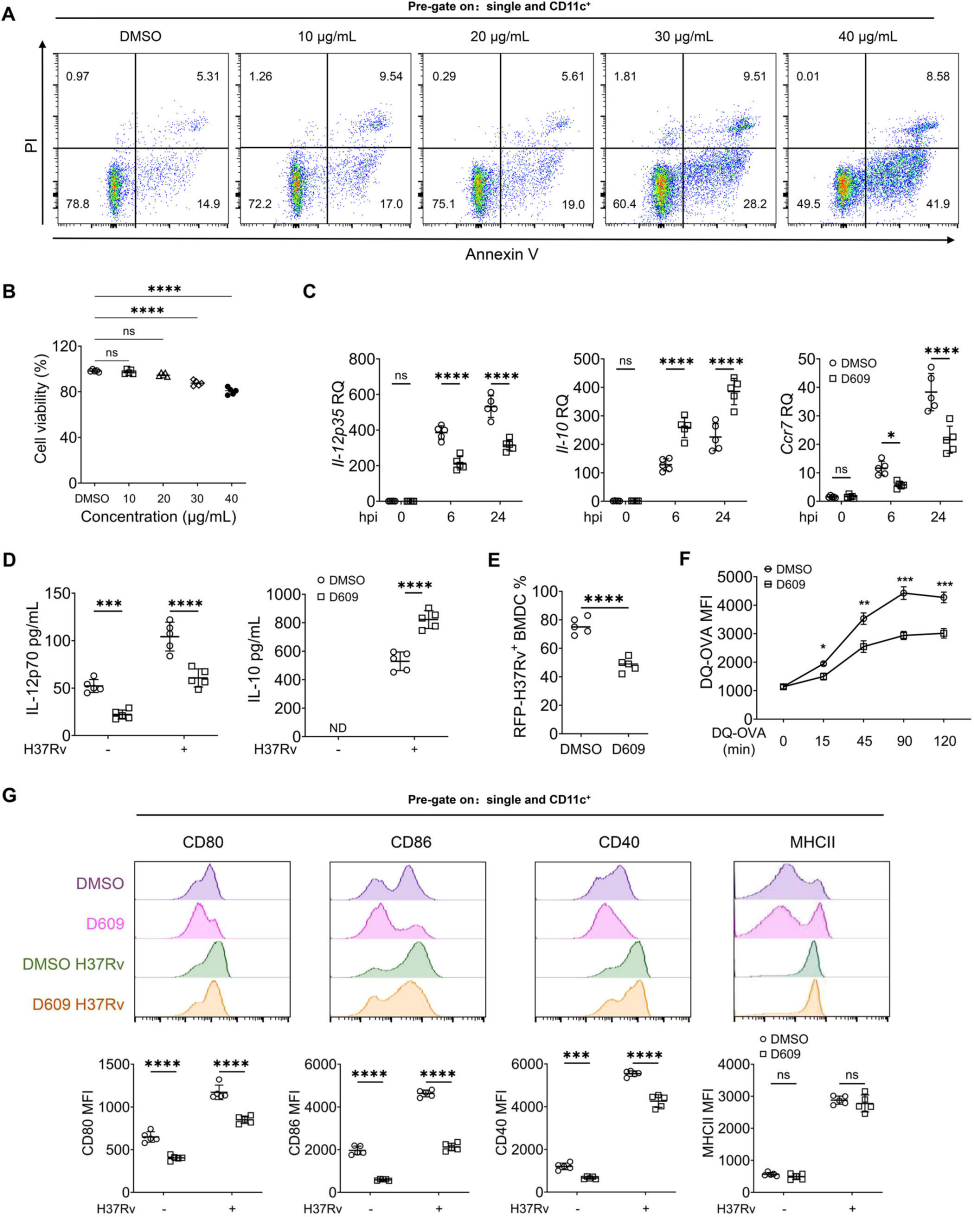
D609 modulates the immune function of BMDCs. (A, B) The effect of D609 on cell viability. The effect of D609 on cell activity was detected by Annexin V/PI (A) and CCK‐8 (B) (*n* = 5). The concentrations of D609 were 10, 20, 30, and 40 μg/mL, respectively. (C) Quantitative real‐time PCR analysis of *Il12‐p35, IL‐10*, and *Ccr7* in D609‐treated BMDCs infected with H37Rv (MOI = 2, *n* = 5). (D) ELISA analysis of IL‐12p70 and IL‐10 in D609‐treated BMDCs infected with H37Rv (MOI = 2, *n* = 5). (E) Flow cytometry analysis of red fluorescence‐positive BMDCs treated with D609, followed by infection with H37Rv‐RFP (MOI = 10, *n* = 5). (F) Flow cytometry analysis of green fluorescence‐positive BMDCs treated with D609, followed by incubation with DQ‐OVA (*n* = 5). (G) Flow cytometry analysis of CD80, CD86, CD40, and MHC‐II expression on the surface of H37Rv‐infected BMDCs at MOI = 2 at 24 hpi (*n* = 5). Statistical analysis was conducted employing a one‐way analysis of variance (ANOVA) test (B), a two‐way ANOVA test with Šidák's post hoc analysis (C, D, F, G), and an unpaired *t*‐test (E). Data are presented as mean ± SD and are representative of at least three experiments with similar observations. *p < 0.05, **p < 0.01, ***p < 0.001, ****p < 0.0001. BMDCs, bone marrow‐derived dendritic cells; CCK‐8, Cell Counting Kit‐8; ELISA, enzyme‐linked immunosorbent assay; SD, standard deviation.

Dendritic cells serve as a bridge connecting the innate immune and adaptive responses, acting as the sole antigen‐presenting cell (APC) that can initiate the activation of naïve T cells. This activation is crucial for the effective control of immunity against tuberculosis (TB) infection [3]. Subsequently, we investigated the influence of D609 on the immunological activity of dendritic cells derived from bone marrow (BMDCs). To exclude the possibility of apoptosis and cell death induced by the drug treatment, we employed Annexin‐V/PI staining (Figure 3A) and the Cell Counting Kit (CCK‐8) Assay (Figure 3B) to assess the impact of D609 on cell viability, taking into account any potential cell toxicity. The results revealed that D609 at a concentration of 20 μg/mL did not elicit significant apoptosis or cell death. We treated BMDCs with D609 and evaluated the expression of cytokines and chemokines associated with BMDC immunological activity after H37Rv infection. Our quantitative real‐time PCR findings revealed that D609 treatment significantly diminished the mRNA levels of *Il‐12p35* and *Ccr7* as compared to the dimethyl sulfoxide (DMSO) group, while concurrently enhancing the transcript levels of the inhibitory cytokine IL‐10 (Figure 3C). To quantify the protein concentrations of IL‐12p70 and IL‐10 in the supernatant, we employed enzyme‐linked immunosorbent assay (ELISA), The results suggested that the changes in protein levels were in line with the alterations observed in mRNA expression (Figure 3D), and the level of reduction of IL‐12p70 was more significant after the infection. In summary, the data unequivocally demonstrated that D609 treatment effectively suppressed the immune response of BMDCs.

### 2.3 D609 Reduces Antigen Phagocytosis and Processing in BMDCs

Dendritic cells, the most potent antigen‐presenting cells, are pivotal in the fight against tuberculosis due to their ability to uptake and process antigens. We employed RFP‐H37Rv and DQ‐OVA, a fluorescently labeled derivative of ovalbumin that emits a verdant fluorescence upon enzymatic cleavage into peptide fragments, to explore the influence of D609 on the phagocytic and antigen processing capabilities of BMDCs. After pretreatment with D609, cells were loaded with RFP‐H37Rv and DQ‐OVA, followed by flow cytometry analysis to compare the results with the DMSO control. We observed a significant reduction in bacterial phagocytosis (Figure 3E) and antigen processing ability (Figure 3F) in D609‐treated BMDCs compared to the DMSO group. These findings suggest that D609 not only affects the secretion of effector molecules by BMDCs but also impairs their ability to phagocytose and process antigens.

The uptake of antigens is intricately linked to the expression of costimulatory molecules. Moreover, the expression of these molecules on antigen‐presenting cells (APCs) plays a crucial role in activating T cells, alongside cytokines. For further investigation, we assessed the expression of surface costimulatory molecules in BMDCs that were pretreated with D609 and subsequently challenged with H37Rv, in comparison to the DMSO control group. D609‐treated BMDCs exhibited a significant reduction in the mean fluorescence intensities of costimulatory molecules, including CD80, CD86, CD40 (Figure 3G), and the differences in the reduced levels of costimulatory molecules were more pronounced after infection. This suggests that D609 diminishes the antigen uptake capacity of BMDCs by suppressing the expression of costimulatory molecules, ultimately affecting the antigen presentation function of activated T cells.

### 2.4 D609 Reduces the Ability of BMDCs to Stimulate Naïve CD4^+^ T Activation

**Figure 4:**
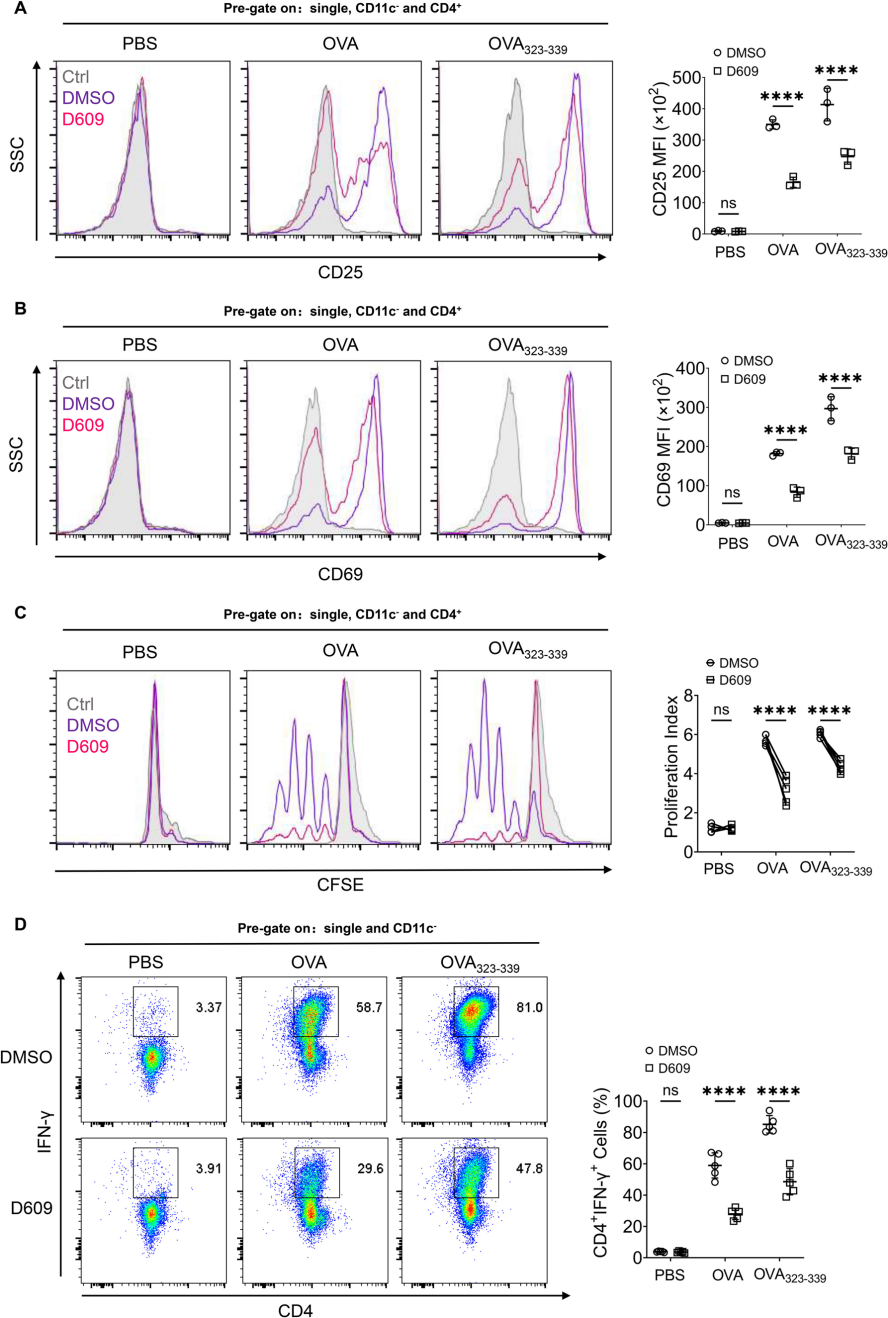
D609 regulates BMDCs‐mediated T‐cell immunity. (A, B) Flow cytometry analysis was performed on the mean fluorescence intensity of activation markers CD25 and CD69 of OT‐II naïve CD4^+^ T cells cocultured with BMDCs loaded with OVA or OVA_323‐339_, CD25 MFI (A), CD69 MFI (B), (*n* = 3). (C) Flow cytometry analysis of T proliferation of CFSE‐stained OT‐II naïve CD4^+^ T cells cocultured with BMDCs loaded with OVA or OVA_323‐339,_ (*n* = 5). (D) Flow cytometry analysis of CD4^+^IFN‐γ^+^ T differentiation of OT‐II naïve CD4^+^ T cells cocultured with BMDCs loaded with OVA or OVA_323‐339,_ (*n* = 5). A two‐way ANOVA with Šidák's post hoc test (A–D) was used for statistical analysis. Data are presented as mean ± SD and are representative of at least three experiments with similar observations. *p < 0.05, **p < 0.01, ***p < 0.001, ****p < 0.0001. ANOVA, analysis of variance; BMDCs, bone marrow‐derived dendritic cells; SD, standard deviation.

Dendritic cells have been documented as bridging the gap between innate and adaptive immunity through their initiation of T cell activation. They possess the exclusive ability to initiate activation of naïve T cells. To investigate the direct modulation of immune function in BMDCs, we proceeded to examine whether BMDCs pretreated with D609 could elicit varying levels of T cell stimulation. To validate this, BMDCs were subjected to pretreatment with D609, loaded with both OVA and OVA_323‐339_, and then cocultured with naïve CD4^+^ T cells from OT‐II mice in a ratio of 1:10. Flow cytometry analysis demonstrated a decrease in the mean fluorescence intensity of activation markers CD25 (Figure 4A) and CD69 (Figure 4B) in CD4^+^ T cells after pretreatment of BMDCs with D609 and subsequent loading with OVA and OVA_323‐339_, in comparison to the DMSO group. These findings suggest that D609‐exposed BMDCs possess the capability to regulate the immune activation of T cells.

### 2.5 D609 Inhibits BMDCs from Stimulating Naïve CD4^+^ T Proliferation and IFN‐γ^+^ T Differentiation

The suppressive effect of D609 on T‐cell activation was observed following pretreatment of BMDCs. Subsequently, we investigated the potential of D609 in modulating the proliferation and differentiation of T cells induced by BMDCs. BMDCs were pretreated with D609 and then cocultured with naïve OT‐II CD4^+^T cells labeled with carboxyfluorescein diacetate succinimidyl este (CFSE) and loaded with OVA and OVA_323‐339_. Flow cytometry analysis revealed that, compared to the DMSO group, BMDCs pretreated with D609 significantly inhibited the proliferation of CFSE‐labeled CD4^+^T cells, indicating a substantial reduction in proliferation (Figure 4C). Additionally, we assessed the proportion of IFN‐γ^+^ T cells in the coculture system, as they possess the capability to selectively eliminate *M. tuberculosis*‐infected cells. Flow cytometry analysis demonstrated a remarkable decrease in the differentiation of CD4^+^ IFN‐γ^+^ T cells in BMDCs pretreated with D609 and loaded with OVA and OVA_323‐339_ (Figure 4D). Our findings elucidate the impact of D609 on both the innate immune activity regulated by BMDCs and the adaptive immune processes stimulated by BMDCs, involving T cells.

## 3 Discussion

During tuberculosis (TB) infection, one or more factors of virulence are indispensable for supporting the survival, growth, and colonization of Mycobacterium tuberculosis in the host. The outcome of the infection is determined by a multitude of factors from both the host and the pathogen [18, 19]. D609 is a well‐established compound with diverse pharmacological properties, as evidenced by numerous studies conducted over the past three decades. This formulation encompasses a wide range of beneficial effects, including its antioxidant [5], antiapoptotic [11], anticholinergic [2], anti‐inflammatory [20], antitumor [21], antiproliferative [22], antiviral [11], and neuroprotective [23] characteristics. These properties can largely be attributed to the inherent ability of D609 to competitively inhibit PC‐PLC and SMS [11], a mechanism proposed to underlie its functionality. PC‐PLC is responsible for the breakdown of membrane phospholipids, generating DAG and IP3. These molecules activate signaling pathways involved in various processes, including the immune response. The role of D609 in *M. tuberculosis* infection was investigated using a mouse model. It has been observed that the administration of D609 results in substantially augmented burdens within the pulmonary and splenic tissues, accompanied by pronounced impairment of the pulmonary tissue and a reduction in inflammatory cytokines. Furthermore, an elevation in the occurrence of multinucleated giant cells (MGCs) has been detected within the spleen. These findings hint at the potential of D609 to impede the host's immune response against tuberculosis by interfering with its crucial substrate, PC‐PLC and SMS.

There are 16 recognized isoforms of phospholipase C (PLC), each distinct in terms of their structure, regulation, and tissue‐specific distribution. PLC serves as a crucial virulence factor for numerous bacteria, impeding the maturation of cytosolic phagosomes, escaping from phagosomes, colonizing tissues, establishing infections, and causing pathogenesis. Several diseases, including cancer and infectious diseases, have been linked to different forms of PLC [17, 24, 25, 26]. PLC can induce cytoskeletal rearrangement in DC2.4 cells, thereby facilitating the entry of *M. tuberculosis* into the intracellular compartment. Nevertheless, the precise impact of phospholipase C (PLC) on the abundance and immune functionality of DCs remains enigmatic. Given the scarcity of relevant research in this field, this present investigation aims to shed light on whether the inhibition of PLC activity by D609 exerts a regulatory influence on the quantity and immune function of DCs. The results of the murine tissue flow assay unveiled that treatment with D609 led to a decrease in the ratio of conventional type 1 DCs (cDC1) to lung tissue and cDC2 in the LNs. The functional consequence of D609‐induced inhibition of PLC activity in bone marrow‐derived DCs (BMDCs) was a reduction in the production of effector molecules, such as IL‐12p35, IL‐10, Ccr7, and surface costimulatory molecules. Furthermore, the outcomes of the H37Rv and DQ‐OVA assays employing fluorescence labeling unequivocally demonstrated that D609 significantly impairs the phagocytic and antigen processing capabilities of DCs.

DCs, as the sole dedicated antigen‐presenting cells capable of stimulating the activation of primary T cells, play a multifaceted role in converting the innate immune response into adaptive immunity during *M. tuberculosis* infection [18, 19, 27]. The diversity within the DC subpopulation allows for altered function, as they interact with other immune cells, *M. tuberculosis*, and its products to enhance host defense mechanisms or promote pathogen evasion [18, 19, 27]. The interaction between DCs and other immune cells contributes to their heterogeneity and altered function. Therefore, gaining a better understanding of the immune responses initiated, promoted, amplified, or suppressed by DCs during *M. tuberculosis* infection can contribute to the development of control measures against tuberculosis, such as host‐directed adjuvant therapies and anti‐TB vaccines. In this investigation, our in *vivo* experiments have unequivocally substantiated the impact of D609 on the abundance of tissue DCs and their distinct subcategories. Moreover, in *vitro* we have demonstrated that D609 not only impinges on the function of BMDCs, but also exerts a profound influence on their capacity to elicit, propagate, and differentiate T‐cells in a coculture milieu comprising BMDCs and OT‐II CD4^+^ T‐cells. These findings strongly imply that D609 not only modulates the innate immune response to TB, but also intricately orchestrates the adaptive immune response.

In conclusion, it can be inferred that D609 not only hampers the host's immune response but also hinders a diverse range of immune functions in dendritic cells (DCs), thereby constraining the efficacy of antituberculosis immunity. Phospholipase C (PLC) may be imperative for DCs to generate chemokines and pro‐inflammatory cytokines, facilitate migration to lymph nodes, and present antigens to T cells, ultimately resulting in T cell activation and serving as scavengers for *Mycobacterium tuberculosis*. Hence, acquiring a more profound comprehension of the regulation of PLC in DCs could unveil potential and innovative targets for the advancement of anti‐TB therapies.

## 4 Methods

### 4.1 Mice

SPF C57BL/6J mice were provided by the Southern Medical University Laboratory Animal Management Centre of Southern Medicine University (Guangzhou, China). OT‐II mice were purchased from Jackson Laboratory (USA) and bred at the Animal Management Centre of Southern Medicine University. The experiment protocol was approved by the Biosafety Management Committee and the Medical Ethics Committee of Southern Medical University.

### 4.2 Preparation of BMDCs and OT‐II CD4^+^ T Cells

Femurs of 6–8‐week female mice were collected and incubated in RPMI1640 medium (Corning Inc., Manassas, VA, USA) containing 20 ng/mL murine GM‐CSF (PeproTech, Rocky Hill, NJ, USA) for 8 days, with half medium replacement every 3 days, to obtain primary BMDCs. OT‐II mice CD4^+^ T cells were negatively separated from spleen cells using MACS (Miltenyi) and cultured in the RPMI 1640 medium supplemented with 10% fetal bovine serum (FBS, Corning Inc.), 2‐ME (Thermo Fisher Scientific Inc., Rockford, IL), and 20 ng/mL IL‐2 (PeproTech) at 37°C with 5% CO_2_.

### 4.3 M. Tuberculosis Culture

Standard *M. tuberculosis* strain H37Rv (ATCC #27294) was cultured in Difco™ Middlebrook 7H9 medium (BD Biosciences, San Jose, CA, USA) supplemented with 1/9 volume of oleic acid‐albumin‐dextrose–catalase (OADC) and 0.05% Tween‐80 (Merck Millipore) at 37°C with 5% CO_2_. For experiments, H37Rv suspension in the mid‐log phase was centrifuged and resuspended in serum‐free RPMI1640 containing 0.05% Tween‐80. After grinding for 30–50 times, the achieved homogenate containing single bacterium was centrifuged again at 1500 × g for 5 min, and the obtained supernatant was detected using the Biophotometer Plus spectrophotometer (Eppendorf, Hamburg, Germany) at an optical density (OD) of 600 nm. The concentration of bacterial suspension with OD 0.207 was regarded to be 4 × 10^6^ colonies/mL.

### 4.4 Animal Experiments

To assess the effects of D609 in anti‐*M. tuberculosis* immune response, C57BL/6 J mice (*n* = 5) were exposed to 10^7^ of H37Rv in the aerosol generation device (Glas‐Col LLC. Terre Haute, IN, USA) for 24 h, Briefly, log‐phase H37Rv cultures were washed twice with 1× PBS and were sonicated to generate single cell suspension. Bacteria were then resuspended in 10 mL 1× PBS at an OD600 of 0.1, and 5 ml of this inoculum was loaded into the inhalation exposure nebulizer. The aerosol unit was programmed to deliver ~100 CFUs per animal, as determined by plating whole lung homogenates from five mice on Middlebrook 7H10 agar for CFU counting within 24 h of infection.

Mice were executed 2‐ and 5‐week post infection. Part of lung tissues were suspended in PBS and grinded into single cells. The supernatants were stored at −80°C for ELISA assays (Multi Sciences (Lianke) Biotech, Co. Ltd. Hangzhou, Zhejiang, China) according to the instructions of the producers. Cells were lysed with 0.2% Triton‐PBS for detecting bacterial loads using CFU assays. Another part of tissues was cut into 5 μm thick of slices following fixation with 4% paraformaldehyde‐PBS and paraffin imbed. H&E staining was used to assess tissue inflammation and damage. In addition, we used a standard assessment method to assess lung injury. In detail, lung injury was scored based on edema, neutrophil infiltration, hemorrhage, bronchiole epithelial desquamation, and hyaline membrane formation, and five visual fields were observed for each slice. The observations were performed in a blinded manner. A score of 0–4 was used to represent the severity of injury: 0 for no damage, 1 for mild damage, 2 for moderate damage, 3 for severe damage, and 4 for very severe damage [28]. In some experiments, Mice were treated with 200 ng of D609 through intraperitoneal injection 1‐week post H37Rv infection.

### 4.5 BMDC Infection

For BMDC infection, H37Rv in Middle brook 7H9 medium (BD Biosciences) with 0.05% Tween‐80% and 10% OADC enrichment (BD Biosciences) were grown to midlogarithmic phase at OD600 of ~0.6. BMDCs were seeded in 12‐well plates at a density of 5.0 × 10^5^ cells per well and precultured in RPMI‐1640 medium supplemented with 10% FBS for 12 h before infection. H37Rv were collected and washed twice in 1× phosphate‐buffered saline (PBS) containing 0.05% Tween‐80 and were then pelleted and thoroughly resuspended using RPMI‐1640 medium with 0.05% Tween‐80. BMDCs were then infected with H37Rv for 1 h at 37°C to allow bacterial entry into cells. Thereafter, the culture media were discarded, and the cells were washed three times with 1× PBS to exclude noninternalized bacteria and were then incubated again with the fresh medium. At each designated time point, the cells and culture supernatants were collected for different analyses.

### 4.6 RNA Extraction and Quantitative Real‐Time Polymerase Chain Reaction (RT‐qPCR)

The total RNA of cells was extracted using the TransZol® Reagent (Transgene Biotech, Beijing, China). Standard agarose gel electrophoresis was used to assess the RNA integrity and the RNA concentration and purity were detected using the NanoDrop 2000 Ultraviolet–Vis Spectrophotometer (Thermo Fisher, Wilmington, DE, USA). Target gene expression was analyzed following reverse transcription of the total RNA using the TransScript One‐Step gDNA Removal and cDNA Synthesis SuperMix kit (Transgene Biotech) and qPCR using TransStart Top Green qPCR SuperMix kit (Transgene Biotech) on the LightCycler96 (Roche, Basel, Switzerland). The expression of *β‐actin* was used as the reference normalization to quantify the target mRNA abundance using the 2^−ΔΔCT^ method. Primer sequences are listed in Table 1.

**Table 1:** Quantitative real‐time polymerase chain reaction (RT‐qPCR) primer sequences.

Gene	Primer sequence
*Il‐12p35*	Forward primer: 5′‐CAATCACGCTACCTCCTCTTTT‐3′
	Reverse primer: 5′‐CAGCAGTGCAGGAATAATGTTTC‐3′
*Il ‐10*	Forward primer: 5′‐GCTCTTACTGACTGGCATGAG‐3′
	Reverse primer: 5′‐ CGCAGCTCTAGGAGCATGTG‐3′
*Ccr7*	Forward primer: 5′‐TGTACGAGTCGGTGTGCTTC‐3′
	Reverse primer: 5′‐GGTAGGTATCCGTCATGGTCTTG‐3′
*β‐actin*	Forward primer: 5′‐ TCAAGATCATTGCTCCTCCTGAG‐3′
	Reverse primer: 5′‐ ACATCTGCTGGAAGGTGGACA‐3′

### 4.7 ELISA Analyses

The lung tissues were suspended in PBS and grinded into single cells centrifuged at 4°C, 12,000 r/min for 5 min, the supernatants were stored at −80°C for ELISA assays. The collected cell supernatants were centrifuged at 4°C, 12,000 r/min for 10 min, then stored at −80°C for ELISA assays. The content in the supernatants was detected by referring to the instructions of the corresponding TNF‐α, IL‐12p70, IL‐10, and IFN‐γ ELISA kits (Multi Sciences (Lianke) Biotech, Co. Ltd. Hangzhou, Zhejiang, China).

### 4.8 CFSE Staining

OT‐II mice CD4^+^ T cells were negatively separated from spleen cells using MACS. Next, 1% bovine serum albumin (BSA)‐phosphate‐buffered saline (PBS) was resuspended to which 5 nM CFSE (Tonbo Biosciences. San Diego, CA, USA) was added and incubated at 37°C to avoid light staining for 20 min, mixed every 5 min, followed by the addition of 5 times volume of 4°C pre‐cooled 10% FBS‐RPMI 1640 and kept on an ice bath for 10 min. Finally, it was centrifuged and resuspended by 10% FBS‐RPMI 1640 with 20 ng/mL IL‐2. Afterward, 2 × 10^5^ cells/well were spread on a 96‐well U‐plate.

### 4.9 BMDC‐T Cell Coculture

BMDCs were spread into 12‐well plates according to 4 × 10^5^ cells/well, rested for 24 h, pretreated with D609 (20 μg/mL) for 2 h, and subsequently loaded with OVA (10 μg/mL) and OVA_323‐339_ (2 ng/mL) for 5 h, respectively. The wells were rinsed with PBS five times, and the BMDCs were collected. According to the ratio of DC:T = 1:10, BMDCs were added to T‐cell wells of 96‐well U‐plates, and T‐cell activation marker CD25, CD69 was detected after 12–16 h of culture, and the ratio of T‐cell proliferation to IFN‐γ^+^ T cells was detected after 72 h.

### 4.10 Flow Cytometry Analysis

To investigated the number of DCs in the lungs, spleens, and LNs of Mtb‐infected mice treated with D609. The mice were intraperitoneally infected with H37Rv for a duration of 1 week. Subsequently, D609 (200 ng) was administered via intraperitoneal injection at intervals of every 3 days. Finally, 2 weeks postinfection, the mice were executed. Single cells achieved from the spleens, the lungs, and LNs were incubated in the 1% FBS‐PBS containing the following antibodies in the dark at 4°C for 30 min before flow cytometry analysis. APC‐cy7‐CD11c (N418; BioLegend), PE‐cy‐7‐MHC‐II (M5/114.15.2; Invitrogen), APC‐CD8a (53‐6.7; TONBO), efluor‐450‐CD11b (M1/70; eBioscience). cDC: CD11c^+^MHC‐II^+^; cDC1: CD11c^+^MHC‐II^+^CD8^+^CD11b^‐^; cDC2: CD11c^+^MHC‐II^+^CD8^‐^CD11b^+^.

To detected apoptosis and cell death induced by the D609, aspirate the cell culture solution into a centrifuge tube, wash the adherent cells once with PBS, and add appropriate amount of trypsin cell digest to digest the cells. Incubate at room temperature until the adherent cells can be blown down by gentle blowing, then aspirate the tryptic digest. Add the collected cell culture solution, gently blow the cells down, transfer to a centrifuge tube, centrifuge at 1000*g* for 5 min, discard the supernatant, collect the cells, gently resuspend the cells with PBS and count. Take 10^5^ resuspended cells, centrifuge at 1000 g for 5 min, discard the supernatant, and add 195 μL Annexin V‐FITC conjugate to gently resuspend the cells. Add 5 μL Annexin V‐FITC, 10 μL propidium iodide (PI) staining solution, and mix gently. Incubate at room temperature away from light for 10–20 min, followed by placing in an ice bath and resuspending the cells every 5 min during incubation. The cells were resuspended every 5 min during the incubation. Subsequently detected using flow cytometry, Annexin V‐FITC was green fluorescent and PI was red fluorescent.

To analyze the phagocytosis activity of BMDC, cells were infected with H37Rv‐RFP for 1 h, washed and fixed with 4% paraformaldehyde then analyzed by flow cytometry.

To investigated the regulation of antigen processing in DCs by D609 using DQ‐OVA (Cat#: D12053, Thermo Fisher Scientific Inc., Rockford, IL). DQ‐OVA (1 μg/mL) was added to cells for 20 min at 37°C (to measure uptake but not processing and is used as a control), the culture media were discarded, and the cells were washed five times with 1×PBS and then incubated again with the fresh medium at 37°C (to measure processing) at indicated time points. At the end of each incubation period, BMDCs were washed and fixed with 4% paraformaldehyde and analyzed by flow cytometry.

To assess the activation of BMDCs infected with H37Rv, cells were collected with trypsinization and incubated in 1% FBS–PBS containing the following antibodies at 4°C in the dark for 30 min: APC‐Cy7‐CD11c (N418; BioLegend), FITC‐CD80 (16‐10A1; TONBO), APC‐CD86 (GL‐1; TONBO), PE‐Cy7‐MHC‐II (M5/114.15.2; Invitrogen), eFluor 450‐CD40 (HM40‐3, Invitrogen). Finally, the cells were centrifuged at 300 g for 5 min, fixed with 4% paraformaldehyde, and subsequently analyzed.

To assess the activation of CD4^+^ T cells, these were cocultured with BMDCs and cocultured with BMDCs loaded OVA (10 ng/mL) and OVA_323‐339_ (2 ng/mL) for 12–16 h, with trypsinization and incubated in 1% FBS–PBS containing the following antibodies at 4°C in the dark for 30 min: Percp‐Cy5.5‐CD4 (GK1.5; eBioscience), APC‐Cy7‐CD11c (N418; BioLegend), PE‐CD25 (PC61.5; TONBO), PE‐Cy7‐CD69(H1.2F3; TONBO). Cells were subsequently centrifuged at 300*g* for 5 min, fixed with 4% paraformaldehyde, and analyzed.

To assess the proliferation of CD4^+^ T cells, these were cocultured with BMDCs, CFSE‐stained CD4^+^ T cells were cocultured with BMDCs loaded OVA (10 ng/mL) and OVA_323‐339_ (2 ng/mL) for 72 h. The cells were fixed permeabilized and incubated in 1% FBS–PBS containing the following antibodies at 4°C in the dark for 1 h: Percp‐Cy5.5‐CD4 (GK1.5; eBioscience), APC‐Cy7‐CD11c (N418; BioLegend), PE‐IFN‐γ (XMG1.2; eBioscience), Cells were finally centrifuged at 300 g for 5 min and analyzed.

The above cells were detected using Attune NxT flow cytometry (Thermo Fisher) and the data were analyzed with the FlowJo 10 software (BD Biosciences).

#### 4.10.1 Statistical Analysis

The representative data of at least three independent experiments are presented as means ± standard deviation (SD). An unpaired two‐tailed *t*‐test was used to compare the difference when two parameters were involved. One‐way ANOVA was used for comparisons of more than two groups. Two‐way analysis of variance (ANOVA) was used to analyze the influence of two independent factors on a response variable and determine the existence of interaction between the two factors on this response variable. Holm–Šídák method was used for post hoc multiple comparisons. *p* < 0.05 indicated a significant difference in treatment groups. *p < 0.05, **p < 0.01, ***p < 0.001, ****p < 0.0001. All statistical analyses were performed using GraphPad Prism 9.4.1 (San Diego, CA).

## Author Contributions

**Honglin Liu:** conceptualization, data curation, formal analysis, writing–original draft. **Huimin Huang:** formal analysis, investigation, methodology. **Zhen Huang:** formal analysis, investigation. **Yingxuan Chen:** formal analysis, investigation. **Deyou Tan:** formal analysis, investigation. **Xiaoni Wang:** formal analysis, investigation. **Xiaoni Pang:** data curation, formal analysis. **Shuwen Chen:** data curation, formal analysis. **Lianhui Liang:** conceptualization, writing–review and editing. **Haihui Yang:** conceptualization, data curation, writing–review and editing.

## Conflicts of Interest

The authors declare no conflicts of interest.

## Data Availability

All data generated or analyzed during this study are included in this published article. The data that support the findings of this study are available on request from the corresponding authors.

## References

[1] S. Bagcchi , “WHO's Global Tuberculosis Report 2022,” Lancet Microbe 4, no. 1 (2023): e20. 36521512 10.1016/S2666-5247(22)00359-7

[2] J. Shao , C. Sun , L. Su , J. Zhao , S. Zhang , and J. Miao , “Phosphatidylcholine‐Specific Phospholipase C/Heat Shock Protein 70 (Hsp70)/transcription Factor B‐Cell Translocation Gene 2 Signaling in Rat Bone Marrow Stromal Cell Differentiation to Cholinergic Neuron‐Like Cells,” International Journal of Biochemistry & Cell Biology 44, no. 12 (2012): 2253–2260. 23000394 10.1016/j.biocel.2012.09.013

[3] H. Kim and S. J. Shin , “Pathological and Protective Roles of Dendritic Cells in *Mycobacterium tuberculosis* Infection: Interaction Between Host Immune Responses and Pathogen Evasion,” Frontiers in Cellular and Infection Microbiology 12 (2022): 891878. 35967869 10.3389/fcimb.2022.891878 PMC9366614

[4] R. M. Adibhatla , J. F. Hatcher , and A. Gusain , “Tricyclodecan‐9‐yl‐xanthogenate (D609) Mechanism of Actions: A Mini‐Review of Literature,” Neurochemical Research 37, no. 4 (2012): 671–679. 22101393 10.1007/s11064-011-0659-z PMC3299863

[5] B. Wang , L. Wang , S. Gu , et al., “D609 Protects Retinal Pigmented Epithelium as a Potential Therapy for Age‐Related Macular Degeneration,” Signal Transduction and Targeted Therapy 5, no. 1 (2020): 20. 32296021 10.1038/s41392-020-0122-1 PMC7054264

[6] H. S. G. Kalluri , A. Gusain , and R. J. Dempsey , “Regulation of Neural Progenitor Cell Proliferation by D609: Potential Role for ERK,” Molecular Neurobiology 47, no. 2 (2013): 782–789. 23275176 10.1007/s12035-012-8390-6

[7] D. G. Walro and K. S. Rosenthal , “The Antiviral Xanthate Compound D609 Inhibits Herpes Simplex Virus Type 1 Replication and Protein Phosphorylation,” Antiviral Research 36, no. 1 (1997): 63–72. 9330762 10.1016/s0166-3542(97)00040-5

[8] D. Milhas , N. Andrieu‐Abadie , T. Levade , H. Benoist , and B. Ségui , “The tricyclodecan‐9‐yl‐xanthogenate D609 Triggers Ceramide Increase and Enhances Fasl‐Induced Caspase‐Dependent and ‐Independent Cell Death in T Lymphocytes,” International Journal of Molecular Sciences 13, no. 7 (2012): 8834–8852. 22942738 10.3390/ijms13078834 PMC3430269

[9] K. Prymas , A. Świątkowska , G. Traczyk , et al., “Sphingomyelin Synthase Activity Affects TRIF‐Dependent Signaling of Toll‐Like Receptor 4 in Cells Stimulated With Lipopolysaccharide,” Biochimica et Biophysica Acta (BBA) ‐ Molecular and Cell Biology of Lipids 1865, no. 2 (2020): 158549. 31678513 10.1016/j.bbalip.2019.158549

[10] F. Zhang , G. Zhao , and Z. Dong , “Phosphatidylcholine‐Specific Phospholipase C and D in Stimulation of RAW264.7 Mouse Macrophage‐Like Cells By Lipopolysaccharide,” International Immunopharmacology 1, no. 7 (2001): 1375–1384. 11460317 10.1016/s1567-5769(01)00069-8

[11] A. H. Bhat , K. B. Dar , A. Khan , et al., “Tricyclodecan‐9‐yl‐Xanthogenate (D609): Mechanism of Action and Pharmacological Applications,” International Journal of Molecular Sciences 23, no. 6 (2022): 3305. 35328726 10.3390/ijms23063305 PMC8954530

[12] N. Perskvist , L. Zheng , and O. Stendahl , “Activation of Human Neutrophils By *Mycobacterium tuberculosis* H37Ra Involves Phospholipase Cγ2, Shc Adapter Protein, and p38 Mitogen‐Activated Protein Kinase,” Journal of Immunology 164, no. 2 (2000): 959–965. 10.4049/jimmunol.164.2.959 10623845

[13] R. Paroha , R. Chourasia , R. Rai , et al., “Host Phospholipase C‐γ1 Impairs Phagocytosis and Killing of Mycobacteria by J774A.1 Murine Macrophages,” Microbiology and Immunology 64, no. 10 (2020): 694–702. 32816349 10.1111/1348-0421.12839

[14] P. A. Assis , M. S. Espíndola , F. W. Paula‐Silva , et al., “ *Mycobacterium tuberculosis* Expressing Phospholipase C Subverts PGE2 Synthesis and Induces Necrosis in Alveolar Macrophages,” BMC Microbiology 14 (2014): 128. 24886263 10.1186/1471-2180-14-128 PMC4057917

[15] R. Paroha , S. K. Chaurasiya , and R. Chourasia , “Phospholipase C‐γ2 Promotes Intracellular Survival of Mycobacteria,” Journal of Cellular Biochemistry 120, no. 4 (2019): 5062–5071. 30317660 10.1002/jcb.27783

[16] H. Liu , Z. Han , L. Chen , et al., “ZNFX1 Promotes AMPK‐Mediated Autophagy against *Mycobacterium tuberculosis* By Stabilizing Prkaa2 mRNA,” JCI Insight 9, no. 1 (2024): e171850. 38016036 10.1172/jci.insight.171850 PMC10906457

[17] A. Sano , H. Sano , T. Iwanaga , and Y. Tohda , “Functional Role of Phosphatidylcholine‐Specific Phospholipase C in Regulating Leukotriene Synthesis and Degranulation in Human Eosinophils,” European Journal of Pharmacology 884 (2020): 173353. 32707189 10.1016/j.ejphar.2020.173353

[18] Q. Chai , L. Wang , C. H. Liu , and B. Ge , “New Insights into the Evasion of Host Innate Immunity by *Mycobacterium tuberculosis* ,” Cellular & Molecular Immunology 17, no. 9 (2020): 901–913. 32728204 10.1038/s41423-020-0502-z PMC7608469

[19] S. Sundararajan and R. Muniyan , “Latent Tuberculosis: Interaction of Virulence Factors in *Mycobacterium tuberculosis* ,” Molecular Biology Reports 48, no. 8 (2021): 6181–6196. 34351540 10.1007/s11033-021-06611-7

[20] D. Zhou , C. M. Lauderback , T. Yu , S. A. Brown , D. A. Butterfield , and J. S. Thompson , “D609 Inhibits Ionizing Radiation‐Induced Oxidative Damage by Acting As a Potent Antioxidant,” Journal of Pharmacology and Experimental Therapeutics 298, no. 1 (2001): 103–109. 11408530

[21] M. E. Maragoudakis , E. Missirlis , G. D. Karakiulakis , M. Sarmonica , M. Bastakis , and N. Tsopanoglou , “Basement Membrane Biosynthesis as a Target for Developing Inhibitors of Angiogenesis With Anti‐Tumor Properties,” Kidney International 43, no. 1 (1993): 147–150. 7679456 10.1038/ki.1993.24

[22] S. W. P. Rees , E. Leung , J. Reynisson , D. Barker , and L. I. Pilkington , “Development of 2‐Morpholino‐N‐hydroxybenzamides as Anti‐Proliferative PC‐PLC Inhibitors,” Bioorganic Chemistry 114 (2021): 105152. 34328856 10.1016/j.bioorg.2021.105152

[23] R. Sultana , S. F. Newman , H. M. Abdul , et al., “Protective Effect of D609 Against Amyloid‐beta1‐42‐induced Oxidative Modification of Neuronal Proteins: Redox Proteomics Study,” Journal of Neuroscience Research 84, no. 2 (2006): 409–417. 16634065 10.1002/jnr.20876

[24] S. P. Diggle and M. Whiteley , “Microbe Profile: *Pseudomonas aeruginosa*: Opportunistic Pathogen and Lab Rat,” Microbiology 166, no. 1 (2020): 30–33. 31597590 10.1099/mic.0.000860 PMC7273324

[25] Y. N. Wu , X. Su , X. Q. Wang , N. N. Liu , and Z. W. Xu , “The Roles of Phospholipase C‐Beta Related Signals in the Proliferation, Metastasis and Angiogenesis of Malignant Tumors, and the Corresponding Protective Measures,” Frontiers in Oncology 13 (2023): 1231875. 37576896 10.3389/fonc.2023.1231875 PMC10419273

[26] J. Ma , X. Zhang , Y. Song , et al., “D609 Inhibition of Phosphatidylcholine‐Specific Phospholipase C Attenuates Prolonged Insulin Stimulation‐Mediated GLUT4 Downregulation in 3T3‐L1 Adipocytes,” Journal of Physiology and Biochemistry 78, no. 2 (2022): 355–363. 35048323 10.1007/s13105-022-00872-x PMC9242966

[27] W. H. Boom , U. E. Schaible , and J. M. Achkar , “The Knowns and Unknowns of Latent *Mycobacterium tuberculosis* Infection,” Journal of Clinical Investigation 131, no. 3 (2021): e136222. 33529162 10.1172/JCI136222 PMC7843221

[28] Q. Zhang , J. Li , H. Zhong , and Y. Xu , “The Mechanism of Nicotinamide on Reducing Acute Lung Injury by Inhibiting Mapk and NF‐κB Signal Pathway,” Molecular Medicine 27, no. 1 (2021): 115. 34544355 10.1186/s10020-021-00376-2 PMC8451170

